# The Emergence of an Urban Mortality Advantage in Brazil: An Assessment of Age and Causes-of-Death Contributions to the Urban–Rural Mortality Gap

**DOI:** 10.1007/s11524-026-01076-0

**Published:** 2026-04-23

**Authors:** José H. C. Monteiro da Silva, Eugenio Paglino, Gabriel M. Borges, Marília R. Nepomuceno

**Affiliations:** 1 Population Studies Center, University of Pennsylvania, Philadelphia, PA USA; 2 Helsinki Institute for Demography and Population Health, Faculty of Social Sciences, University of Helsinki, Helsinki, Finland; 3 Max Planck – University of Helsinki Center for Social Inequalities in Population Health, Helsinki, Finland; 4 Latin American and Caribbean Demographic Centre, Population Division of the United Nations Economic Commission for Latin America and the Caribbean, Santiago, Chile; 5 Max Planck Institute for Demographic Research, Rostock, Germany

**Keywords:** Mortality differentials, Adult mortality, Urban–rural mortality, Brazil

## Abstract

Brazil has historically seen higher mortality among urban residents compared to rural counterparts, despite better self-reported health among urban residents and better access to healthcare services. However, recent trends in these urban–rural mortality differentials and the specific causes of death contributing to them have not been well documented. We address this gap by analyzing trends in adult life expectancy in Brazil’s five macro-regions from 2006 to 2023. We decompose changes in the life expectancy gap by age, sex, and cause of death. Our findings reveal an emerging urban advantage in male life expectancy in Brazil, beginning in the mid-2000s and continuing until the start of the COVID-19 pandemic. For females, a small initial urban survival penalty had shifted to an urban advantage by 2019. The North and Northeast regions showed larger urban penalties for both sexes than the southern regions. For males, the emerging urban life expectancy advantage was primarily driven by a more rapid decrease in mortality from external causes among urban residents aged 20–39. For females, the urban advantage was fueled by relative gains in mortality from neoplasms, respiratory diseases, and external causes. The COVID-19 pandemic initially widened the urban mortality penalty or narrowed the urban advantage in 2020 due to higher COVID-19 mortality in urban areas. However, this trend reversed from 2021 to 2023, as the urban penalty in COVID-19 mortality turned into an urban advantage, reversing the relative gains seen in rural areas.

**Supplementary Information:**

The online version contains supplementary material available at 10.1007/s11524-026-01076-0.

## Introduction

Similarly to other Latin American countries, Brazil experienced rapid mortality declines starting in the 1930 s [1, 2]. This process was accompanied by public health interventions such as the implementation of vaccination campaigns and the expansion of access to improved sanitation and clean water, which mostly targeted infectious diseases and led to a sharp decline in under-five mortality. All of these developments occurred in the backdrop of a fast urbanization of the country [3–6].

Improvements in health and mortality and the development of the healthcare infrastructure did not, however, occur uniformly across the country, and marked regional and urban–rural inequalities persist [6, 7]. Brazil experienced a fast mortality decline, with a marked shift from communicable to non-communicable diseases and a sharp reduction of infant mortality [8]. This epidemiological transition, however, deviated from the pattern documented for high-income countries [9] and its progression showed substantial subnational variation. For instance, although life expectancy in richer and poorer states has converged over the long term, there were periods of divergence, due particularly to changes in mortality from external causes [7]. States from the North and Northeast regions, historically characterized by lower income, higher shares of rural population, and lower life expectancy, continue to lag behind the Central-West, Southeast, and South regions. In recent years, Southern states have experienced larger gains in life expectancy driven by declines in non-communicable mortality, whereas Northern states have seen more limited gains in part due to persistently high mortality from external causes [10].

These regional variations are reflected at other geographic scales, notably in the disparities between urban and rural areas in Brazil. Rural residents have lower socioeconomic status and report worse health than their urban counterparts [11, 12]. Rural residents also face more constraints in accessing healthcare services, especially among the elderly [13]. Despite these health and socioeconomic disadvantages, an urban mortality penalty has been documented in Brazil at least since the 1970 s [14]. Subsequent studies that evaluated urban–rural mortality differentials confirmed the existence of an urban penalty at adult and older ages [15, 16], though not in under-five mortality [17, 18]. Other research, however, found an urban advantage in life expectancy at birth for large cities and metropolitan areas of the country, in comparison to medium-sized cities and small towns in the period 2000–2010 [19]. Subnational variation in urban–rural survival differentials has not been explored.

This work addresses two gaps that remain in the literature. First, we extend the temporal scope of previous studies by looking at the evolution of urban–rural survival differentials past 2010 and at the effect of the COVID–19 pandemic. Second, we explore the interplay between regional differences in mortality and urban–rural mortality differentials, since national patterns can potentially mask substantial subnational variation. We use data from the vital statistics system and from national censuses to estimate mortality rates and life expectancies between ages 20 and 85 for rural and urban areas and by region in Brazil. Finally, relying on age and cause-of-death decompositions of changes in the urban–rural gap in life expectancy, we examine how mortality at different ages and from different causes of death contributed to the observed trends in urban–rural survival differentials.

## Data and Methods

### Data

Deaths counts for urban and rural areas by region (North, Northeast, Central-West, Southeast, South, and all regions combined), year (2006–2023), 5-year age group (20–24, 25–29, …, 80–84), sex, and causes of death were obtained from publicly available microdata retrieved from the Mortality Information System of the Brazilian Ministry of Health (SIM, from Portuguese *Sistema de Informações sobre Mortalidade*). We chose 2006 as the starting point for our analysis because the proportion of deaths classified as ill-defined was particularly high in the early 2000 s, especially in the North and Northeast regions. We then decided to restrict our analysis to years in which all regions had at maximum 15% of ill-defined causes of death—more details on the prevalence of ill-defined causes of death can be found in the Supplementary Material 3. Similar concerns about data quality led us to limit the age range to 20–85 for multiple reasons. First, we limited our analysis to age 85 to avoid distortions in mortality rates caused by age misreporting mainly in the form of age exaggeration among the oldest old [20–22]. Second, we do not include younger age groups because under-five and adolescent mortality are shaped by different processes compared to adulthood [23]. This would require a separate analysis. Moreover, data quality issues related to the completeness of infant mortality are more pronounced in rural areas [24].

To classify municipalities into rural and urban, we adopt the definition of urban concentrations given by the IBGE [25]. This classification differentiates small cities close to large metropolitan areas from more isolated municipalities with potentially limited access to resources and public healthcare services. Municipalities located within an urban concentration according to IBGE were classified as urban. We adopt the classification for municipalities of 2022 and assume it was the same for 2010 and 2000—under this classification, about 12% of the municipalities, which represent 62% of the total population in 2022, are classified as urban (additional details in the Supplementary Material 1). We grouped causes of death into eight groups: circulatory diseases, communicable diseases (excluding COVID-19), external causes, metabolic diseases, neoplasms, respiratory diseases, COVID-19, and all other causes (ICD10 codes for each group are reported in Table S2.1 in Supplementary Material 2). We redistribute deaths assigned to ill-defined causes of death proportionally to the remaining causes by age and sex. We further adjust death counts according to completeness levels by state separately for urban and rural areas (additional details on our approach and on the data can be found in Supplementary Material 3).

### Constructing Life Expectancies

We estimate corresponding person-years of exposures by age, sex, and state based on the official population counts from the national censuses of 2000, 2010, and 2022 conducted by the Brazilian Institute of Geography and Statistics (IBGE, from Portuguese *Instituto Brasileiro de Geografia e Estatística*)—all of which had August 1 st as the reference date—following standard demographic methods [27]. We first estimate mid-year urban and rural populations (on July 1^st^) in intercensal years by state, age, and sex assuming constant growth between subsequent censuses. Then, we compute yearly proportions of the population living in urban and rural areas for each state, age, and sex combination. As a third step, we apply these proportions to redistribute the official population estimates and projections, updated by IBGE in 2024 [28], which are available yearly at the state level but not distinguish between rural and urban populations. Through this procedure, we ensure that population estimates by state, age, and sex in every year equal those in the IBGE estimates while being able to separately estimate urban and rural populations. The IBGE’s updated estimates are preferable to those taken directly from censuses because they rely on the results of post-enumeration surveys conducted after census rounds and account for census under-reporting. Finally, we estimate annual person-years of exposure in urban and rural areas by state, age, and sex by assuming linear growth within each year.

We calculate age-specific mortality rates for ages 20 to 85 by 5-year age groups, region (*r*), year (*t*), sex (*s*), cause of death (*c*), and area type (*a*) as

$${}_{5}{M}_{x}^{r, t,s,c,a}=\frac{{ }_{5}{D}_{x}^{r, t,s,c,a}}{{ }_{5}{P}_{x}^{r, t,s,c,a}}$$

where *x* refers to the age at the beginning of the 5-year age interval, the numerator *D* correspond to death counts, and the denominator *P* to the population exposures. We then calculate life expectancies between ages 20 and 85, $e(\mathrm{20,85})$, following standard life table calculation approaches and assuming that life table deaths occur in the middle of each age interval [27]. We call $e(\mathrm{20,85})$ adult life expectancy.

### Decomposing Changes in the Urban–Rural Gap in Life Expectancy

Further, we express the urban–rural gap (${G}^{{r},{t},{s}}$) in adult life expectancy for each region *r*, year *t*, and sex *s* as a function of age- and cause-specific mortality rates.

$${G}^{{r},{t},{s}}=e{\left(\mathrm{20,85}\right)}^{{r},{t},{s},{urban}}-e{\left(\mathrm{20,85}\right)}^{{r},{t},{s},{rural}}=f\left({ }_{5}{M}_{{x}}^{{r},{t},{s},{c},{urban}},{ }_{5}{M}_{{x}}^{{r},{t},{s},{c},{rural}}\right)$$

A positive gap (${G}^{{r},{t},{s}}>0$) indicates an urban survival advantage and a negative gap (${G}^{{r},{t},{s}}<0$) represents an urban penalty.

Relying on the line integral decomposition method and on its assumption that age- and cause-specific mortality rates change gradually in time [29], we decompose the change in the adult life expectancy gap$\Delta {G}^{{r},{s}}\left({t}_{1},{t}_{2}\right)$ between successive years in terms of age-, cause-, and area-type- specific components $k_{x,c,a},$ 

$$\Delta {G}^{{r},{s}}\left({t}_{1},{t}_{2}\right) ={G}^{{r},{{t}}_{1},{s}}-{G}^{{r},{{t}}_{2},{s}}={\sum }_{{x}}{\sum }_{{c}}{\sum }_{{a}}{k}_{{x},{c},{a}}$$

The generic component ${k}_{{x},{c},{a}}$ represents the contribution of changes in the mortality rates for age group $x$, cause of death $c$, and area type (rural or urban) $a$ to the change in the gap between subsequent years. To simplify the exposition, we perform the decomposition across successive years and sum the results across selected periods (2006–2012, 2012–2019, 2019–2020, 2020–2021, 2021–2022, 2022–2023, and 2006–2023).

### Content of the Supplementary Materials

We included additional details on the construction of urban and rural population estimates in Supplementary Material 1. We report detailed ICD-10 codes included in each cause-of-death group in Supplementary Material 2. We also visualize trends in age-standardized cause-specific mortality rates in Supplementary Material 2. Supplementary Material 3 contains detailed information on the completeness of vital statistics and the adjustments we performed to reduce the likelihood of undercounting deaths. Finally, Supplementary Material 4 contains a sensitivity analysis by using a different urban–rural classification scheme based on municipality population size (urban if above 50,000 residents and rural otherwise). The replication materials and R codes of our analyses are available at the following GitHub repository https://github.com/josehcms/urban-rural-LEGap-BR.

## Results

### National Trends in Life Expectancy in Rural and Urban Areas 2006–2023

**Fig. 1:**
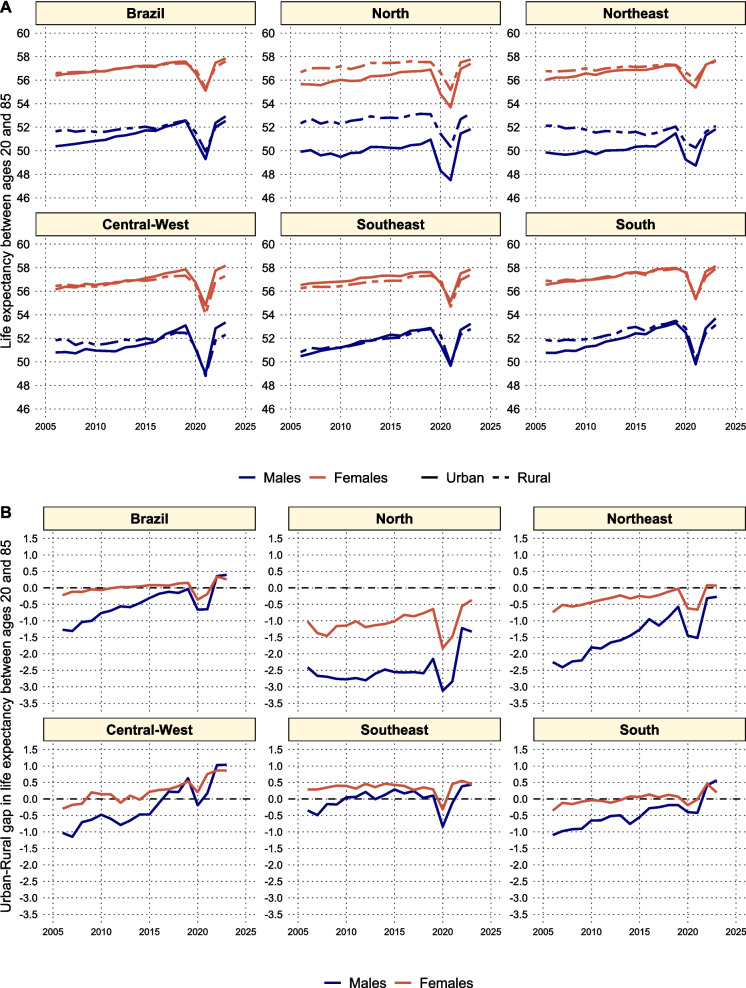
**A** Life expectancy between ages 20 and 85 by area, region, and sex. Brazil, 2006–2023. **B** Urban–rural gap in life expectancy between ages 20 and 85 by area, region, and sex. Brazil, 2006–2023

In 2006, urban areas in Brazil experienced a 0.2-year penalty in adult life expectancy among females and a more sizeable 1.3-year penalty among males (Table 1 and Fig. 1B). Between 2006 and 2019, as female adult life expectancy grew from 56.4 to 57.6 in urban areas and from 56.6 to 57.4 in rural areas, the small urban survival penalty turned into a small survival advantage. Males experienced a similar trajectory, with adult life expectancy rising from 50.4 to 52.5 in urban areas and from 51.7 to 52.6 in rural areas, and the urban survival penalty practically vanishing (Fig. 1A and Table 1). Overall, between 2006 and 2019, female adult life expectancy saw an average annual increase of 0.09 years in urban areas and 0.06 years in rural areas. Male adult life expectancy increased faster than female adult life expectancy in both urban (average annual increase 0.16 years) and rural areas (0.07 years).

**Table 1:** Life expectancy between ages 20 and 85 by area, region, and sex. Brazil, 2006–2023

	Males				Females			
	Urban	Rural	Total	Gap	Urban	Rural	Total	Gap
North								
2006	49.9	52.3	51.2	− 2.4	55.7	56.7	56.1	− 1.0
2012	49.8	52.7	51.3	− 2.8	56.0	57.2	56.5	− 1.2
2019	51.0	53.1	52.0	− 2.2	56.9	57.5	57.2	− 0.6
2020	48.3	51.4	49.8	− 3.1	54.8	56.6	55.6	− 1.8
2021	47.5	50.3	48.9	− 2.8	53.7	55.2	54.3	− 1.5
2022	51.5	52.7	52.1	− 1.2	56.9	57.5	57.2	− 0.6
2023	51.8	53.2	52.5	− 1.3	57.4	57.8	57.5	− 0.4
Northeast								
2006	49.9	52.1	51.2	− 2.3	56.0	56.8	56.4	− 0.7
2012	50.0	51.7	51.0	− 1.7	56.7	57.0	56.9	− 0.3
2019	51.5	52.1	51.8	− 0.6	57.3	57.3	57.3	0.0
2020	49.3	50.7	50.1	− 1.5	56.1	56.7	56.4	− 0.6
2021	48.7	50.3	49.6	− 1.5	55.4	56.0	55.7	− 0.7
2022	51.3	51.6	51.5	− 0.3	57.3	57.2	57.3	0.1
2023	51.8	52.1	52.0	− 0.3	57.7	57.6	57.7	0.1
Central-West								
2006	50.8	51.8	51.3	− 1.0	56.2	56.4	56.3	− 0.3
2012	50.9	51.7	51.2	− 0.8	56.7	56.8	56.7	− 0.1
2019	53.1	52.5	52.9	0.6	57.9	57.3	57.7	0.5
2020	51.1	51.3	51.2	− 0.2	56.7	56.5	56.7	0.2
2021	49.0	48.8	49.0	0.2	54.8	54.0	54.5	0.8
2022	52.9	51.8	52.5	1.0	57.8	56.9	57.5	0.9
2023	53.3	52.3	53.0	1.0	58.2	57.3	57.9	0.9
Southeast								
2006	50.5	50.8	50.6	− 0.3	56.5	56.2	56.5	0.3
2012	51.8	51.5	51.8	0.2	57.1	56.7	57.1	0.5
2019	52.9	52.8	52.9	0.1	57.6	57.3	57.6	0.3
2020	51.4	52.3	51.7	− 0.8	56.7	57.0	56.8	− 0.3
2021	49.7	49.8	49.7	− 0.1	55.1	54.7	55.1	0.5
2022	52.7	52.3	52.7	0.4	57.5	57.0	57.4	0.6
2023	53.2	52.8	53.1	0.4	57.8	57.4	57.8	0.5
South								
2006	50.8	51.9	51.3	− 1.1	56.5	56.9	56.7	− 0.4
2012	51.7	52.2	52.0	− 0.5	57.1	57.2	57.2	− 0.1
2019	53.3	53.5	53.4	− 0.2	58.0	57.9	58.0	0.1
2020	52.5	52.9	52.7	− 0.4	57.6	57.8	57.7	− 0.2
2021	49.8	50.2	50.0	− 0.4	55.3	55.3	55.4	0.0
2022	52.8	52.4	52.7	0.4	57.7	57.2	57.5	0.5
2023	53.7	53.1	53.5	0.6	58.1	57.9	58.1	0.2
Brazil								
2006	50.4	51.7	50.9	− 1.3	56.4	56.6	56.5	− 0.2
2012	51.2	51.8	51.5	− 0.6	57.0	56.9	57.0	0.0
2019	52.5	52.6	52.6	0.0	57.6	57.4	57.5	0.2
2020	50.9	51.6	51.2	− 0.7	56.6	57.0	56.7	− 0.4
2021	49.3	49.9	49.6	− 0.6	55.1	55.3	55.2	− 0.2
2022	52.4	52.0	52.3	0.4	57.5	57.1	57.4	0.3
2023	52.9	52.5	52.8	0.4	57.9	57.6	57.8	0.3

Between 2019 and 2020, the first year of the COVID-19 pandemic, adult life expectancy declines in urban areas (females 1.0 years, males 1.6 years) were larger than in rural areas (females 0.5 years, males 1.0 years), and the urban survival penalty grew. However, the additional adult life expectancy declines from 2020 to 2021 were of similar magnitude in urban and rural areas, and the subsequent recovery in 2022 and 2023 was faster in urban areas. By 2023, female adult life expectancy was 0.3 years higher than in 2019 in urban areas and 0.2 years higher in rural areas. Similarly, male adult life expectancy was 0.4 years higher than in 2019 in urban areas but just about the same in rural areas. As a consequence, urban areas had gained a longevity advantage by 2023 and enjoyed a 0.3-year female adult life expectancy and a 0.4-year male adult life expectancy advantage (Fig. 1B and Table 1).

### Regional Trends in Life Expectancy in Rural and Urban Areas 2006–2023

In 2006, the national urban life expectancy penalty extended to all Brazilian regions for both sexes with the single exception of female life expectancy in the Southeast (Table 1 and Fig. 1B). However, the urban survival penalty was much larger in the Northern regions—North, females 1.0 year, males 2.4; Northeast, females 0.7 years, males 2.3 years—than in the Central-West—females 0.3 years, males 1.0 year—and in the Southern regions—Southeast, females −0.3 years, males 0.3 years; South, females 0.4 year, males 1.1 years. This regional variation persisted even as the urban life expectancy penalty narrowed, and by 2019, the North experienced a sizeable urban life expectancy penalty—females 0.6 years, males 2.2—while urban areas had caught up or surpassed rural areas in the rest of the country. Across all regions and for both sexes, the emergence of an urban life expectancy advantage resulted from larger life expectancy gains in urban than rural areas. The North represents an exception to this pattern because rural and urban areas experienced similar gains in life expectancy between 2006 and 2019—females 1.1 and 0.8 years for urban and rural areas, respectively, and males 1.2 and 0.8 years in urban and rural areas, respectively.

In 2020, urban areas experienced larger life expectancy declines than rural areas across all regions and for both sexes. Both the urban declines—North, females 2.1 years, males 2.6 years; Northeast, females 1.2 years, males 2.2 years—and the rural ones—North, females 0.9 years, males 1.7 years; Northeast, females 0.6 years, males 1.4 years—were largest in the Northern regions. The urban life expectancy penalty increased similarly in all regions—between 0.6 and 1.2 years for females and between 0.8 and 1.0 years for males—except in the Central-West for females and in the South for both sexes, where increases were more contained—Central-West, females 0.3 years; South, females 0.3 years, males 0.2 years. The further life expectancy declines between 2020 and 2021 were similar in rural and urban areas for both sexes but were larger in the Central-West and in the Southern regions than in the Northern regions. All regions experienced large life expectancy gains in 2022, with larger gains in urban than rural areas. By 2023, urban and rural life expectancies had recovered to 2019 levels, but urban areas experienced larger cumulative gains. Urban life expectancy grew similarly across regions—between 0.1 and 0.5 years for females and between 0.2 and 0.9 years for males. Gains in rural life expectancy were also similar across regions—between 0.1 and 0.3 for females, and between 0.0 and 0.1 for males—except in the South and in the Central-West, where male life expectancy declined (South, males −0.4 years decline; and Central-West, males −0.2 years decline), and female life expectancy stayed about the same (South, females 0.01 years increase; and Central-West, females −0.02 years decline).

### Age- and Cause-Specific Contributions to the Evolution of the Urban–Rural Gap in Adult Life Expectancy

**Fig. 2:**
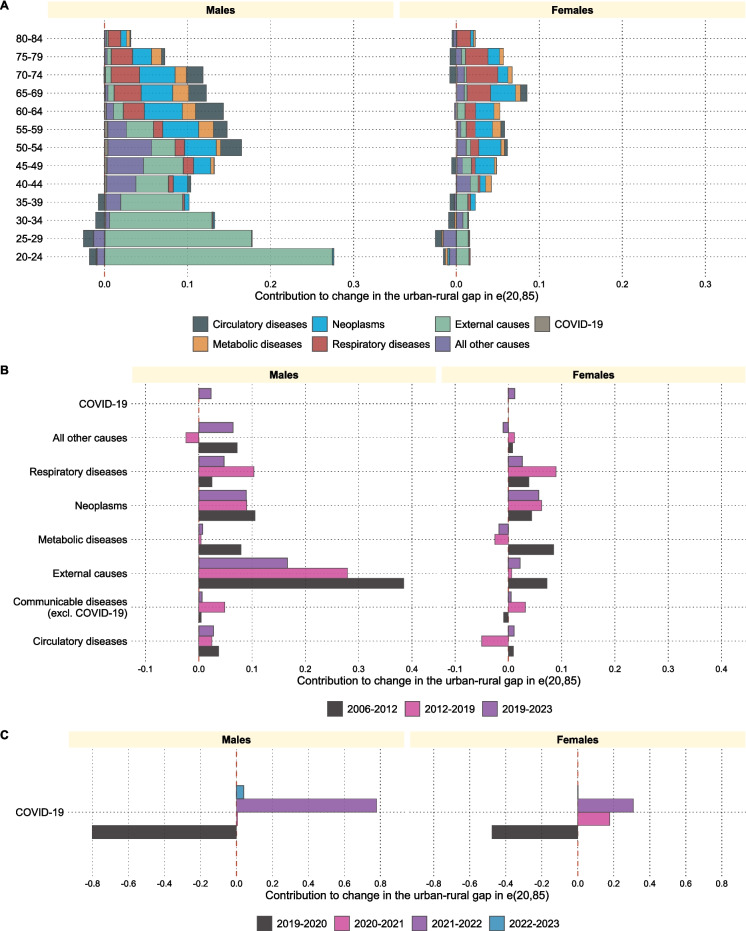
**A** Age- and cause-specific contributions to changes in the urban–rural gap in life expectancy between ages 20 and 85, 2006–2023 by sex. Brazil, 2006–2023. **B** Cause-specific contributions to changes in the urban–rural gap in life expectancy between ages 20 and 85 by period and sex. Brazil, 2006–2023. **C** COVID-19-specific contributions to changes in the urban–rural gap in life expectancy between ages 20 and 85 by year and sex. Brazil, 2019–2023

In Brazil, between 2006 and 2023, the urban–rural gap in adult life expectancy for females shifted from a 0.2-year urban penalty to a 0.3-year urban advantage. Figure 2 shows that for females, faster mortality declines in urban than rural areas in middle adulthood (40–64) and older adulthood (65–84) were responsible for the shift from an urban mortality penalty to an urban advantage between 2006 and 2023 (Fig. 2 and Table 2). Mortality changes in young adulthood (20–39) had only a marginal (3%) contribution.

**Table 2:** Cause-specific contributions to changes in the urban–rural life expectancy gap (2006–2023)

	Males				Females			
Panel A: Age-specific contributions to changes in the urban–rural life expectancy gap (2006–2023)								
Age	2006–2012	2012–2019	2019–2023	2006–2023	2006–2012	2012–2019	2019–2023	2006–2023
20–24	0.06	0.11	0.09	0.26	0.00	− 0.01	0.00	0.00
v25–29	0.04	0.06	0.05	0.15	− 0.01	0.00	0.00	− 0.01
30–34	0.06	0.04	0.02	0.12	0.01	− 0.01	0.00	0.01
35–39	0.09	0.01	0.00	0.09	0.00	0.01	0.01	0.02
40–44	0.07	0.05	− 0.01	0.10	0.04	0.00	0.00	0.04
45–49	0.08	0.02	0.03	0.13	0.01	0.03	0.00	0.04
50–54	0.09	0.07	0.01	0.17	0.02	0.02	0.02	0.06
55–59	0.05	0.05	0.05	0.15	0.02	0.02	0.02	0.06
60–64	0.05	0.02	0.07	0.14	0.03	− 0.01	0.03	0.05
65–69	0.03	0.03	0.06	0.12	0.04	0.02	0.02	0.09
70–74	0.06	0.02	0.03	0.12	0.05	0.02	− 0.01	0.06
75–79	0.02	0.04	0.01	0.07	0.03	0.03	− 0.01	0.05
80–84	0.01	0.01	0.01	0.03	0.01	0.00	0.00	0.02
Total	0.71	0.53	0.43	1.66	0.25	0.13	0.11	0.48
Panel B: Cause-specific contributions to changes in the urban–rural life expectancy gap (2006–2023)								
Cause of death	2006–2012	2012–2019	2019–2023	2006–2023	2006–2012	2012–2019	2019–2023	2006–2023
Circulatory diseases	0.04	0.02	0.03	0.09	0.01	− 0.05	0.01	− 0.03
Communicable diseases (excl. COVID-19)	0.00	0.05	0.01	0.07	− 0.01	0.03	0.01	0.03
External causes	0.38	0.28	0.17	0.83	0.07	0.01	0.02	0.10
Metabolic diseases	0.08	0.00	0.01	0.09	0.09	− 0.03	− 0.02	0.04
Neoplasms	0.11	0.09	0.09	0.28	0.04	0.06	0.06	0.16
Respiratory diseases	0.02	0.10	0.05	0.18	0.04	0.09	0.03	0.15
All other causes	0.07	− 0.02	0.06	0.11	0.01	0.01	− 0.01	0.01
COVID-19	0.00	0.00	0.02	0.02	0.00	0.00	0.01	0.01
Total	0.71	0.53	0.43	1.66	0.25	0.13	0.11	0.48
Panel C: Contributions of COVID-19 mortality to changes in the urban–rural life expectancy gap (2019–2023)								
Cause of death	2019–2020	2020–2021	2021–2022	2022–2023	2019–2020	2020–2021	2021–2022	2022–2023
COVID-19	− 0.80	0.01	0.78	0.04	− 0.48	0.18	0.31	0.00

For males, the adult urban–rural life expectancy gap shifted from a 1.3-year urban penalty to a 0.4-year advantage, but the changes in the age pattern driving this shift were markedly different compared to females (Fig. 2 and Table 2). Among males, faster mortality declines in urban than rural areas in young adulthood contributed 38% of the overall decline in the urban survival penalty. Disproportionately faster urban mortality declines in middle (42%) and older (21%) adulthood also contributed but to a lesser extent than for female mortality (Fig. 2 and Table 2).

The observed patterns of age-specific contributions to changes in the adult urban–rural life expectancy gaps reflect underlying differences in the distribution of causes of death between urban and rural areas and between men and women (Fig. 2 and Table 2). For females, declines in mortality from respiratory diseases and neoplasms accounted for 66% of the reversal of the urban penalty, with external deaths contributing another 21%. For males, external deaths alone contributed about 50% to the urban penalty decline, with neoplasms and respiratory diseases jointly contributing 28%. For both females (2%) and males (10%), circulatory and metabolic diseases contributed surprisingly little to changes in the urban mortality penalty. This modest contribution, despite their significant share of overall mortality (in 2019, females 40%, males 35%), is partially explained by offsetting trends: while urban mortality declines for these causes were faster in middle and older adulthood, rural declines were more rapid in young adulthood (Fig. 2 and Table 2). Cause-specific age-standardized mortality rates in urban and rural areas by region and sex are reported in Fig. S2.1 in Supplementary Material 2.

### Differential COVID-19 Mortality in Urban and Rural Areas 2019–2023

In 2020, COVID-19 mortality was substantially higher in urban than rural areas, and COVID-19 alone contributed 0.48 years to the female urban life expectancy penalty and 0.80 years among males. However, the urban penalty in COVID-19 mortality disappeared in 2021 and turned into an urban advantage in 2022 contributing to narrowing the urban adult life expectancy penalty by 0.31 years among females and 0.78 years among males and erasing the gains in 2020. By 2023, COVID-19 mortality was no longer contributing to survival differences between rural and urban areas. At the same time, other causes of death continued to contribute to the reversing of the urban survival penalty during the COVID-19 pandemic and between 2019 and 2023, the urban survival advantage grew by 0.11 years among females and by 0.43 years among males.

## Discussion

Past research has documented an urban survival penalty in Brazil at least since the 1970 s [14] which persisted at least to 2010 [15, 16] in spite of the fact that urban residents are better off both in terms of socioeconomic status and access to health care [11, 12]. In this study, we evaluate mortality trends in urban and rural areas over 2006–2023, incorporating data from the most recent decade and the COVID-19 pandemic, and disentangle the contributions of causes of death and age groups to changes in urban–rural adult life expectancy differentials. We also analyze these trends at the subnational level, revealing substantial regional variation.

Our findings show that the initial urban penalty in adult life expectancy observed in the middle 2000 s turned into an urban advantage over the last two decades for both males and females in the country as a whole, and in the Central–West, Southeast, and South regions. In the North and Northeast, the two regions that started from the largest urban survival penalty, the gap between rural and urban areas narrowed throughout the period of analysis but did not turn into an urban survival advantage. For females, the reversal of the urban survival penalty was mostly driven by middle and older adult ages and by faster mortality declines in urban areas compared to rural areas. Neoplasms and respiratory diseases each accounted for approximately one third of this trend, while external deaths contributed about 21%. Among males, external deaths alone were responsible for half of the urban survival penalty decline, with neoplasms and respiratory diseases contributing close to a third to the total decline. Despite representing a large share of overall mortality, circulatory and metabolic diseases had a surprisingly small contribution to the reversal of the urban survival penalty, partly because faster declines in urban than rural areas above age 40 were offset by larger declines in rural than urban areas at younger ages.

There are multiple potential explanations for the trends we observe. First, the initial gap in adult life expectancy can be explained not only by lower mortality from external causes in rural areas in the middle 2000 s (as a result of high urban violence), but also by differences in lifestyle and behaviors that are risk factors for chronic conditions. Evidence from other contexts supports this interpretation: in Indonesia, for example, the reported rural adult mortality advantage was found to be mediated by the lower BMI of rural residents in relation to their urban counterparts [30]. Further, the faster decline of circulatory diseases mortality in urban areas can be associated with a convergence of dietary patterns of rural and urban areas. In addition, previous works have shown that in more recent periods, the dietary patterns of rural and urban areas have been converging more and more toward higher consumption of industrialized and processed foods with lower nutritional value [31–33], and the consumption of ultra-processed foods has been linked to a higher risk of circulatory diseases [34, 35].

Second, as Brazil transitions from a regime of higher mortality from communicable diseases to higher mortality from non-communicable conditions, access to more complex and specialized health services and professionals starts to play a major role in defining trends in mortality [9]. In this context, the faster mortality decline in urban areas and the slower or stagnating mortality improvements in rural areas observed for neoplasms, circulatory, metabolic, and respiratory diseases can be linked to the limited access to more specialized health care medical professionals in rural areas [13]. In more isolated areas, delayed diagnosis of cancers or chronic conditions may result in premature mortality. Even though the development and expansion of the Brazilian Unified Health System, and especially of primary care programs such as the Family Health Strategy, was key for improving access to health services among the more isolated areas and to the more vulnerable socioeconomic groups [36, 37], the increased demand for more complex health services can be hard to meet if there is difficulty in accessing these services either due to socioeconomic or logistic hindrances. Conditions such as cancer and circulatory diseases require more specialized and complex care and medical technology, which, along with medical personnel, are more concentrated in urban areas. Therefore, we believe that persistent urban–rural inequalities in the quality of, and access to, more complex health services provided by the Unified Health System and national health programs could partially explain the observed trends in the mortality gap.

Third, the high contributions of external causes of death reflect efforts to reduce homicide deaths in Brazilian major cities. It may also indicate shifts in the regional distribution of violence in the country, with organized crime spreading to rural areas [38, 39]. More studies at the subnational level are needed to understand how external causes are evolving in the country.

The reversal of the urban survival penalty into an urban survival advantage observed in Brazil is not unique, having also been documented in other contexts. In the United States, for example, an initial urban mortality penalty was later reversed to a higher mortality in non-metropolitan areas, and this advantage of metropolitan areas has been continuously increasing through time [40, 41]. In low- and middle-income countries, there is a higher heterogeneity in terms of urban mortality bias, and it seems to be related to the stage of epidemiological transition and level of mortality of the country [19, 42].

### Limitations

This study comes with some limitations. First, the mortality information system of Brazil lacks the standard definition of urban and rural areas used by IBGE in the census; therefore, we needed to adopt a standard classification across these two data sources at the municipality level. Different studies and institutions have adopted different classifications, and this may explain the differences in results we observe. For instance, some previous works adopted a definition from the IBGE [18, 42] while others used city size [17]. We used the most recent classification of urban concentrations from IBGE because it considers the socioeconomic integration of territories. We conducted a sensitivity analysis considering city size as a criterion, defining rural areas as municipalities with less than 50,000 inhabitants and urban areas as municipalities with more than 50,000 inhabitants, and we obtained very similar results, with no changes in our main conclusions (see Supplementary Material 4). Second, although we improved on previous studies by leveraging new data on completeness levels and a new method to differentially adjust urban and rural mortality rates for underregistration of deaths, residual urban–rural differentials in death registration completeness could bias our results, especially in the Northern areas in the middle 2000 s, where undercoverage in rural areas was likely very high. Therefore, we are more confident about data quality after 2010, when death registration was mostly complete in most states of the country [26, 43. Third, cause-of-death classification is a challenging process and can result in errors. This means that differences in the quality of cause-of-death data between urban and rural areas may affect our findings, particularly in the Northern region. For example, the stagnation in mortality from neoplasms and metabolic diseases in rural areas that we documented could be a data artifact due to improvements in data quality in rural areas over time. Our estimates of the contribution of COVID-19 to changes in the urban–rural mortality gap during 2020–2023 may also be sensitive to data limitations. These estimates are likely underestimated because in Brazil, as in many other countries, excess deaths during the pandemic exceeded COVID-19 deaths [44], with some analyses suggesting at least partial misattribution of COVID-19 deaths to other causes of death [45].

### Conclusions

This study demonstrates that Brazil’s long-standing urban mortality penalty reversed to an urban survival advantage for adults over the past two decades, with marked regional variation. Our findings illuminate that this shift was predominantly a consequence of greater mortality reductions in urban areas across specific age groups and causes of death, including external causes for men and neoplasms and respiratory diseases for women. The urban survival advantage that emerged in the Central-West, Southeast, and South regions, together with the persistence of an urban penalty in the North and Northeast, underscores that national-level health progress can mask persistent, regionally concentrated health inequities. To foster more uniform improvements in population health, policy efforts must prioritize strengthening the capacity of rural health systems to deliver specialized care for non-communicable diseases, which are increasingly becoming major mortality drivers as the Brazilian population ages. Additionally, understanding the diverging trends in mortality from circulatory and metabolic diseases—with faster urban declines at older ages but larger rural declines at younger ages—requires further investigation and targeted interventions that address lifestyle factors and access to early detection and management therapies across the urban–rural continuum. Ultimately, achieving health equity in Brazil necessitates a granular approach to health policy, acknowledging and proactively addressing the distinct mortality profiles and healthcare needs of its diverse urban and rural populations.

## Supplementary Information

Below is the link to the electronic supplementary material.

ESM 1 (DOCX 33.9 MB)

ESM 2 (DOCX 356 KB)

ESM 3 (DOCX 451 KB)

ESM 4 (DOCX 887 KB)
