# Supplementary material for: The Emergence of an Urban Mortality Advantage in Brazil: An Assessment of Age and Causes-of-Death Contributions to the Urban–Rural Mortality Gap
Source: J Urban Health. 2026 Apr 23;103(3):587–99. doi: 10.1007/s11524-026-01076-0 (PMC13315069; doi:10.1007/s11524-026-01076-0)
Supplement: Supplementary file 1 — (DOCX 33.9 MB) [file 11524_2026_1076_MOESM1_ESM.docx]

**Supplemental material 1: Brazilian population by urbanicity**

**Urban-rural classification**

In our work we use the IBGE classification of urban concentrations proposed in 2016 and adjusted to the Brazilian geography of the 2022 census [1]. Even though the census data (used to calculate the denominators of mortality rates) have the information about the urbanicity of each household, the public microdata from the SIM does not contain information about the urbanicity of the residence of the deceased. The lowest geographic level that we can get from the SIM microdata is the municipality of residence of the deceased. To harmonize this information between data sources, we then used a criterion that classifies the whole municipality as urban or rural. For that, we rely on the IBGE classification of municipalities as part of *urban concentrations*, which are defined by *population arrangements* (groups of two or more municipalities that are highly integrated regarding the socioeconomic activities of their populations) with more than 100,000 inhabitants or by single isolated municipalities with at least 100,000 inhabitants [2]. Hence, if a municipality is within an *urban concentration* we classified it as urban, and as rural, otherwise.

Under this classification, Figure S1.1 reports which municipalities were then classified as urban or as rural in each region. In Table S1.1 we show the total population and the share of urban population of Brazil and its regions in census years (2000, 2010, and 2022).

**Figure S1.1** Classification of municipalities according to the proposed urban-rural criteria, 2022.


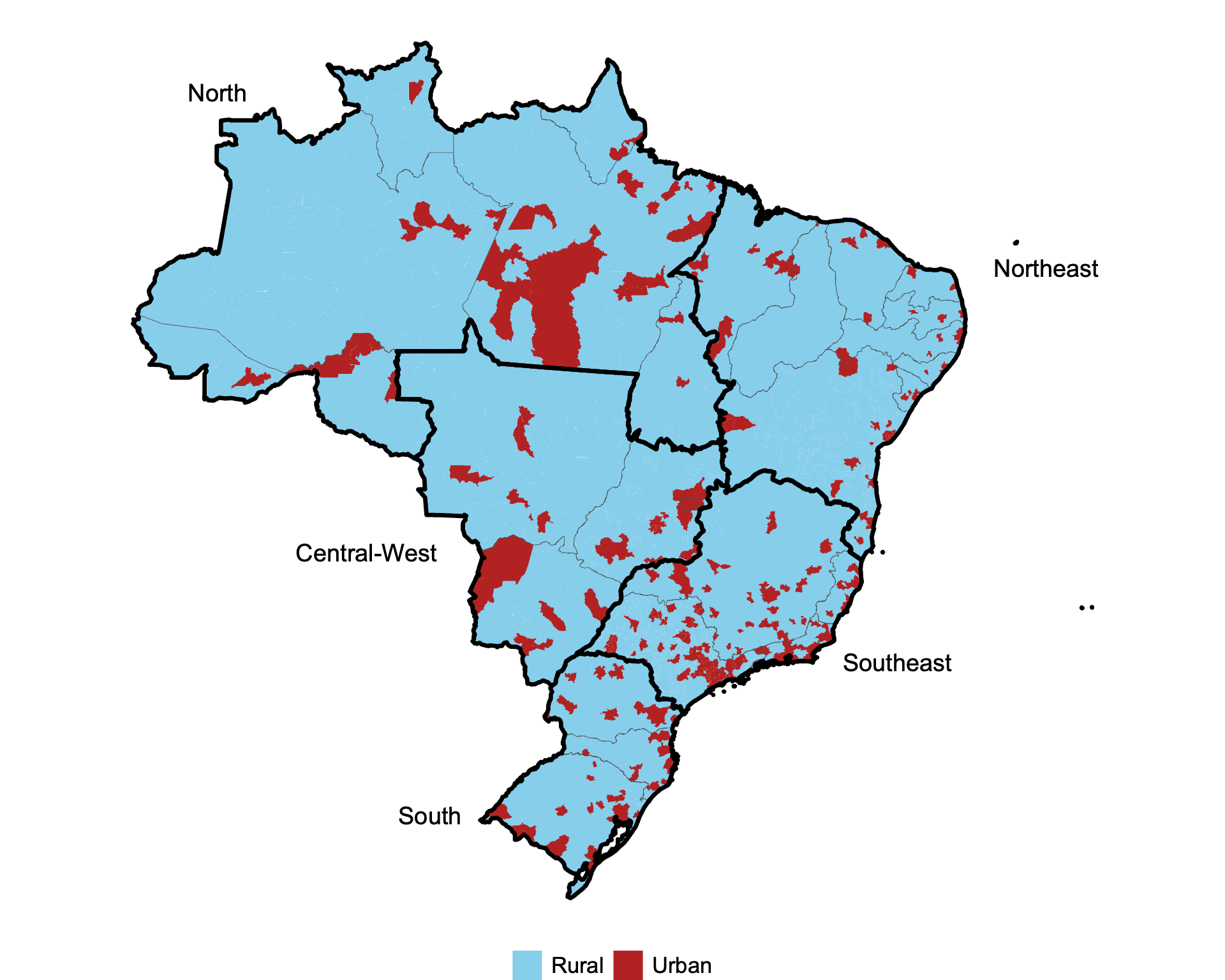


**Table S1.1** Total (adjusted) population and percentage of urban population of Brazil and regions in census years.

| **Region** | **2000** | **2010** | **2022** |
| --- | --- | --- | --- |
| **Brazil** |  |  |  |
| **Population** | 174,695,935 | 194,749,329 | 210,862,983 |
| **% Urban** | 60.0 | 61.3 | 62.3 |
| **North** |  |  |  |
| **Population** | 13,340,960 | 16,158,154 | 18,403,491 |
| **% Urban** | 48.7 | 49.7 | 50.7 |
| **Northeast** |  |  |  |
| **Population** | 48,974,858 | 53,692,489 | 56,848,401 |
| **% Urban** | 42.4 | 44.1 | 45.3 |
| **Southeast** |  |  |  |
| **Population** | 74,758,366 | 82,575,202 | 88,178,052 |
| **% Urban** | 74.9 | 75.7 | 76.2 |
| **South** |  |  |  |
| **Population** | 25,503,013 | 27,910,392 | 30,704,015 |
| **% Urban** | 56.3 | 58.4 | 59.8 |
| **Central-West** |  |  |  |
| **Population** | 12,118,738 | 14,413,092 | 16,729,024 |
| **% Urban** | 61.4 | 63.2 | 64.7 |

# **References**

1 IBGE. *Arranjos populacionais e concentrações urbanas do Brasil*. 2nd edn. Rio de Janeiro: Instituto Brasileiro de Geografia e Estatística - IBGE 2016.

2 IBGE, editor. *Quadro geográfico de referência para produção, análise e disseminação de estatísticas*. 2nd edn. Rio de Janeiro, RJ: Instituto Brasileiro de Geografia e Estatística - IBGE 2022.
