# Supplementary material for: The Emergence of an Urban Mortality Advantage in Brazil: An Assessment of Age and Causes-of-Death Contributions to the Urban–Rural Mortality Gap
Source: J Urban Health. 2026 Apr 23;103(3):587–99. doi: 10.1007/s11524-026-01076-0 (PMC13315069; doi:10.1007/s11524-026-01076-0)

# **Supplemental Material 2: Causes of death**

**Classification of causes of death**

We use the ICD-10 chapters to build our own classification of causes of death considering the groups of causes that respond for the largest share of mortality in Brazil. Table S2.1 reports the adopted classification. We summarize the ICD10 codes into 9 groups of causes of death: circulatory diseases, communicable diseases (excluding COVID-19), COVID-19, external causes, metabolic diseases, neoplasms, respiratory diseases, ill-defined causes, and all other causes. We further redistribute the ill-defined conditions proportionately by age and sex within each municipality.

**Age-standardized mortality rates by causes of death**

We use death counts by macro-region (r), year (t), area of residence (a), sex (s), five-year age groups (ages x to x+5), and causes of death (c), and corresponding exposures to construct age-specific mortality rates by for ages 20 and to 84 (20-24, 25-29, …, 85+). We use${}_{5}M_{x}^{r,t,a,s,c}$ to denote the age-specific mortality rate for ages x to x+5 for region r, in year t, of area a, by sex s, and for cause c.

Using this set of age-specific mortality rates and a standard age-distribution ${}_{5}P_{x}$ (the Brazilian population distribution for both sexes in 2022), we construct age-standardized mortality rates (ASMR) for each area by region, year, cause of death, and sex: $ASMR^{r,t,a,s,c}=\sum_{x} {}_{5}M_{x}^{r,t,a,s,c}\cdot{}_{5}P_{x}$.

**Table S2.1:** Classification used in the analysis of causes of death.

| **ICD10 Chapter** | **ICD10 Code** | **Description** | **Classification** |
| --- | --- | --- | --- |
| I | A00-B99 (except B342) | Certain infectious and parasitic diseases | Communicable diseases (excl. COVID-19) |
| I | B342 | COVID-19 | COVID-19 |
| II | C00-D48 | Neoplasms | Neoplasms |
| III | D50-D89 | Diseases of the blood and blood-forming organs and certain disorders involving the immune mechanism | All other causes |
| IV | E00-E90 | Endocrine, nutritional and metabolic diseases | Metabolic diseases |
| V | F00-F99 | Mental and behavioral disorders | All other causes |
| VI | G00-G99 | Diseases of the nervous system | All other causes |
| VII | H00-H59 | Diseases of the eye and adnexa | All other causes |
| VIII | H60-H95 | Diseases of the ear and mastoid process | All other causes |
| XIX | I00-I99 | Diseases of the circulatory system | Circulatory diseases |
| X | J00-J99 | Diseases of the respiratory system | Respiratory diseases |
| XI | K00-K93 | Diseases of the digestive system | All other causes |
| XII | L00-L99 | Diseases of the skin and subcutaneous tissue | All other causes |
| XIII | M00-M99 | Diseases of the musculoskeletal system and connective tissue | All other causes |
| XIV | N00-N99 | Diseases of the genitourinary system | All other causes |
| XV | O00-O99 | Pregnancy, childbirth and the puerperium | All other causes |
| XVI | P00-P96 | Certain conditions originating in the perinatal period | All other causes |
| XVII | Q00-Q99 | Congenital malformations, deformations and chromosomal abnormalities | All other causes |
| XVIII | R00-R99 | Symptoms, signs and abnormal clinical and laboratory findings, not elsewhere classified | Symptoms, signs, and ill-defined conditions |
| XIX | S00-T98 | Injury, poisoning and certain other consequences of external causes | External causes |
| XX | V01-Y98 | External causes of morbidity and mortality | External causes |
| XXI | Z00-Z99 | Factors influencing health status and contact with health services | All other causes |

**Figure S2.1.** Age-standardized mortality rates (ASMR) by cause of death and by urbanicity, Brazil 2006-2023. The Brazilian population distribution of both sexes of 2022 was used as the standard for calculating adult mortality age-standardized mortality rates.


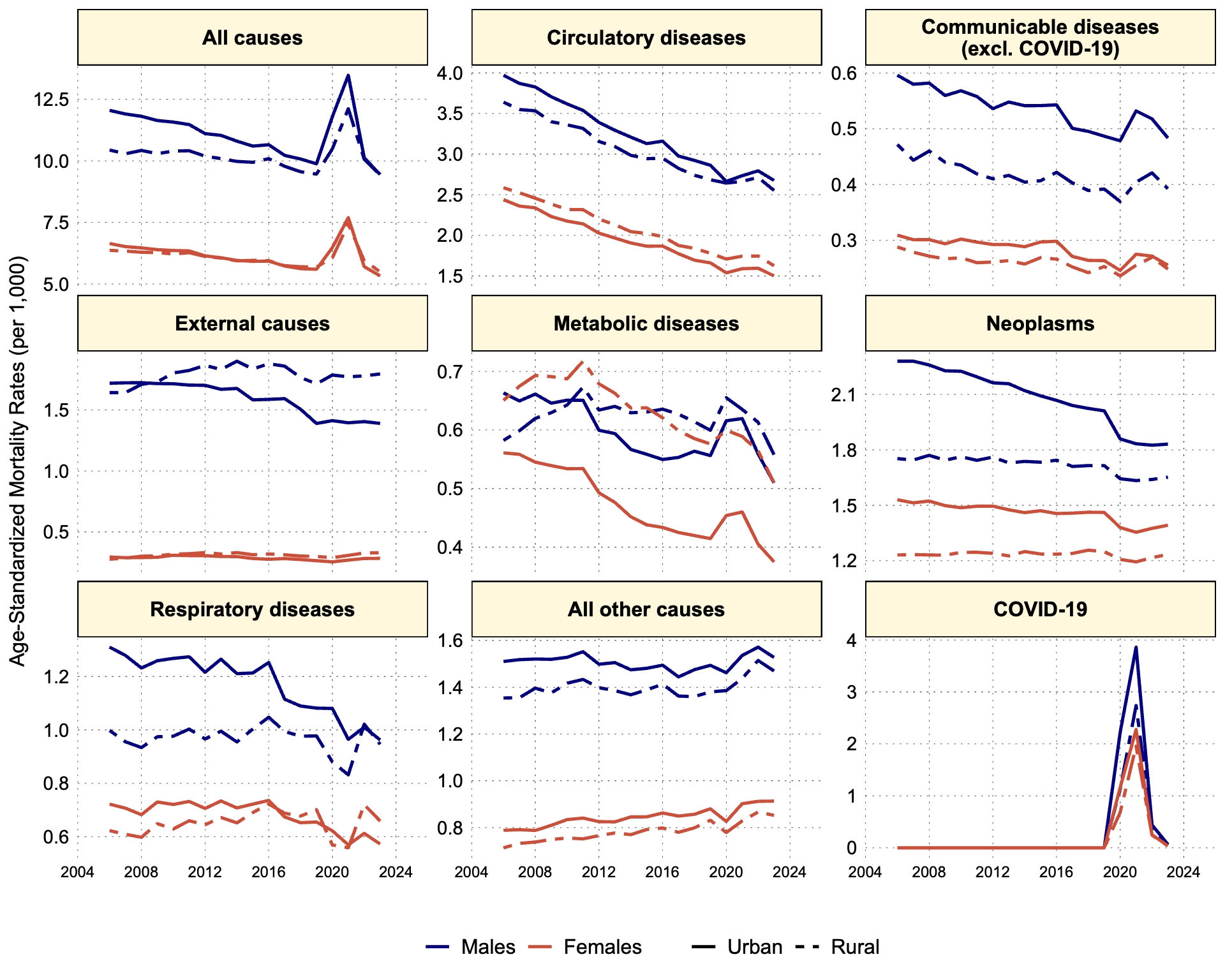

Supplement: Supplementary file 2 — (DOCX 356 KB) [file 11524_2026_1076_MOESM2_ESM.docx]
