# Supplementary material for: The Emergence of an Urban Mortality Advantage in Brazil: An Assessment of Age and Causes-of-Death Contributions to the Urban–Rural Mortality Gap
Source: J Urban Health. 2026 Apr 23;103(3):587–99. doi: 10.1007/s11524-026-01076-0 (PMC13315069; doi:10.1007/s11524-026-01076-0)
Supplement: Supplementary file 3 — (DOCX 451 KB) [file 11524_2026_1076_MOESM3_ESM.docx]

# **Supplemental Material 3: Completeness of vital statistics by urbanicity**

The SIM (Mortality Information System) data from the Ministry of Health is considered of good quality at the national level, especially in more recent years. However, substantial state-level variation persists in the completeness of death registration and in the extent and quality of the demographic information and cause-of-death coding [1–4]. To account for the potential under-reporting of deaths in the SIM dataset, we leveraged the *busca ativa* (proactive search) estimates of completeness from the Ministry of Health [5,6]. This source provides state-level estimates of completeness of registration for the SIM dataset from 2000 to 2022. We assume that for 2023 the completeness was the same as in 2022. Due to the lack of disaggregated estimates at the municipality level, we use the state-level estimates of completeness of death records from the SIM and we assume that these levels for urban and rural areas within states are related to the data quality of the mortality information (for which we use the information on ill-defined causes of deaths as a proxy) –– details on the estimation of these completeness levels are provided in the supplementary materials. Due to the unavailability of disaggregated estimates by sex, age and causes of death, we further assume that these urban and rural completeness levels are the same for both sexes, causes of deaths, and across age groups. In the following paragraphs we describe in detail the approach used to adjust the completeness of death counts information.

The Brazilian Ministry of Health publishes every year an updated series of levels of death registration completeness of the SIM by state based on a proactive search procedure^[[1]](#footnote-1)^. These procedure selects a sample of municipalities and inspects official and unofficial sources, such as cemeteries and funeral homes, in order to find deaths that were not reported and then estimate correction factors to adjust the number of observed deaths [5,7]. Considering that deaths occurring in rural and isolated areas are more likely to be underreported in the vital statistics systems [5,8], we further adapt these state level estimates of death registration completeness to account for the urban-rural differentials. The IBGE provides municipality level completeness estimates for both the SIM and the civil registration system using a capture-recapture methodology [9], however these series starts on 2015, when the completeness of the SIM was much higher than in the early 2000s, especially in the Northern areas of the country [4]. Then, we rely on the proactive search estimates to further adjust the overall completeness for each corresponding area.

We assume that the completeness of death registration in a given urban-rural area is proportional to the proportion of deaths that are not ill-defined, i.e., deaths not coded in chapter XVIII of ICD10, relying on the correlation that exists between death registration completeness and data quality of the death reports [1]. Figure S3.1 shows that the proportion of ill-defined causes has been declining in Brazil and its regions, but it is consistently higher in rural areas.

The completeness of death registration for a given urban-rural area a is given by the quotient between the reported ($R^{a}$) and the true number ($T^{a}$) of deaths.

$$C^{rural}=\frac{R^{rural}}{T^{rural}} (1)$$

$$C^{urban}=\frac{R^{urban}}{T^{urban}} (2)$$

We then write the completeness for each area $C^{a}$ a as a function of the proportion of deaths properly coded $P^{a}$ multiplied by a factor $k^{a}$, relying on our previously stated assumption of proportionality between data quality and death registration completeness.

$$C^{rural}=k^{rural}\times P^{rural} (3)$$

$$C^{urban}=k^{urban}\times P^{urban} (4)$$

Then, we can relate $C^{rural}$ and $C^{urban}$:

$$\frac{C^{rural}}{C^{urban}}=\frac{k^{rural}\times P^{rural}}{k^{urban}\times P^{urban}}= K\frac{P^{rural}}{P^{urban}} (5)$$

Using the available completeness estimates for the SIM at the municipality level provided by the IBGE using the capture-recapture methodology^[[2]](#footnote-2)^ and the proportion of deaths that are not ill-defined from the period between 2015-2022, we can estimate $k^{rural}$ and $k^{urban}$ using equations 3 and 4, assuming that these values are constant across the period of analysis. This results in $k^{rural} =1.03$ and $k^{urban} =1.02$. Therefore, the factor K from equation 5 is approximately 1, and thus, we can write:

$$\frac{C^{rural}}{C^{urban}}\approx\frac{P^{rural}}{P^{urban}}=F \left( 6 \right)$$

Further, we need to make sure that the death at the state level correspond to the sum of deaths from rural and urban areas. Then, the overall state-level completeness, which is the value provided by the proactive search, can be written as:

$$C=\frac{R}{T}\Rightarrow T=\frac{R}{C} (7)$$

Then,

$$T=T^{urban}+T^{rural}=\frac{R^{urban}}{C^{urban}}+\frac{R^{rural}}{C^{rural}}=\frac{R}{C} (8).$$

Using the relationship given by equation 6,

$$T=\frac{R^{urban}}{C^{urban}}+\frac{R^{rural}}{F\times C^{urban}}=\frac{R}{C} \left( 9 \right).$$

Rearranging the terms in equation 9 finally brings us to:

$$C^{urban}=\frac{C}{R}\times\left( R^{urban}+\frac{R^{rural}}{F} \right)\left( 10 \right).$$

All values from the right-hand side of the equation are available, which allows us for estimating $C^{urban}$, and consequently $C^{rural}$ using equation 6. For values of completeness higher than 1, we set them to 1.

The estimated completeness values by year, urbanicity and region can be seen on Figure S3.2. We restricted our analysis to years 2006-2023 because the share of ill-defined deaths was too high before 2005 as can be seen in Figure S3.1, therefore, we decided to keep only years in which we had less than 15% ill-defined deaths in the national-level rural areas.

**References**

1 Lima EEC de, Queiroz BL. Evolution of the deaths registry system in Brazil: associations with changes in the mortality profile, under-registration of death counts, and ill-defined causes of death. *Cad Saúde Pública*. 2014;30:1721–30. doi: https://doi.org/10.1590/0102-311X00131113

2 Martins Soares Filho A, Lima RBD, Merchan-Hamann E, *et al.* Improving the quality of external cause of death data in Brazil: evaluation and validation of a new form to investigate garbage codes. *Cad Saúde Pública*. 2023;39:e00097222. doi: 10.1590/0102-311xen097222

3 Paes NA. Avaliação da cobertura dos registros de óbitos dos estados brasileiros em 2000. *Rev Saúde Pública*. 2005;39:882–90. doi: https://doi.org/10.1590/S0034-89102005000600003

4 Queiroz BL, Freire FHMDA, Gonzaga MR, *et al.* Estimativas do grau de cobertura e da mortalidade adulta (45q15) para as unidades da federação no Brasil entre 1980 e 2010. *Rev bras epidemiol*. 2017;20:21–33. doi: 10.1590/1980-5497201700050003

5 Almeida W da S de, Szwarcwald CL, Frias PG de, *et al.* Capturing deaths not informed to the Ministry of Health: proactive search of deaths in Brazilian municipalities. *Rev bras epidemiol*. 2017;20:200–11. doi: https://doi.org/10.1590/1980-5497201700020002

6 Brazilian Ministry of Health. Indicadores de cobertura que utilizam a metodologia do Busca Ativa: Cobertura de informações de óbito. 2024.

7 Szwarcwald CL, de Frias PG, Júnior PRB deSouza, *et al.* Correction of vital statistics based on a proactive search of deaths and live births: evidence from a study of the North and Northeast regions of Brazil. *Population Health Metrics*. 2014;12:16. doi: 10.1186/1478-7954-12-16

8 Arriaga EE. Rural-Urban Mortality in Developing Countries: An Index for Detecting Rural Underregistration. *Demography*. 1967;4:98–107. doi: 10.2307/2060354

9 Costa LFL, De Mesquita Silva Montenegro M, Rabello Neto DDL, *et al.* Estimating completeness of national and subnational death reporting in Brazil: application of record linkage methods. *Popul Health Metrics*. 2020;18:22. doi: 10.1186/s12963-020-00223-2

**Figure S3.1:** Share of deaths classified in chapter XVIII (Symptoms, signs, and ill-defined conditions) of ICD-10 in SIM by year, Brazil and regions, 2000-2023. The yellow line marks year 2006.


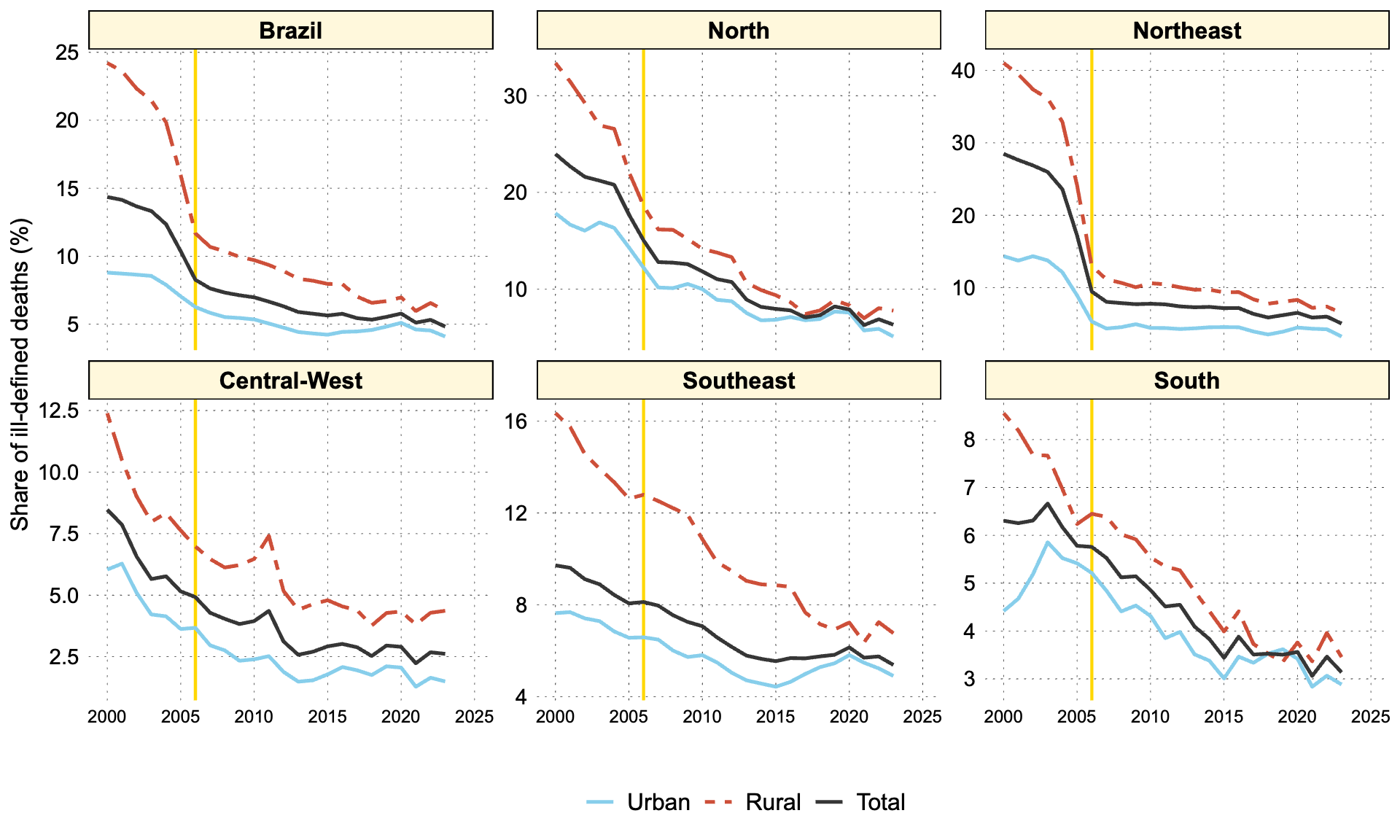


**Figure S3.2:** Estimated death registration completeness levels by urbanicity, Brazil and regions, 2006-2023.

**
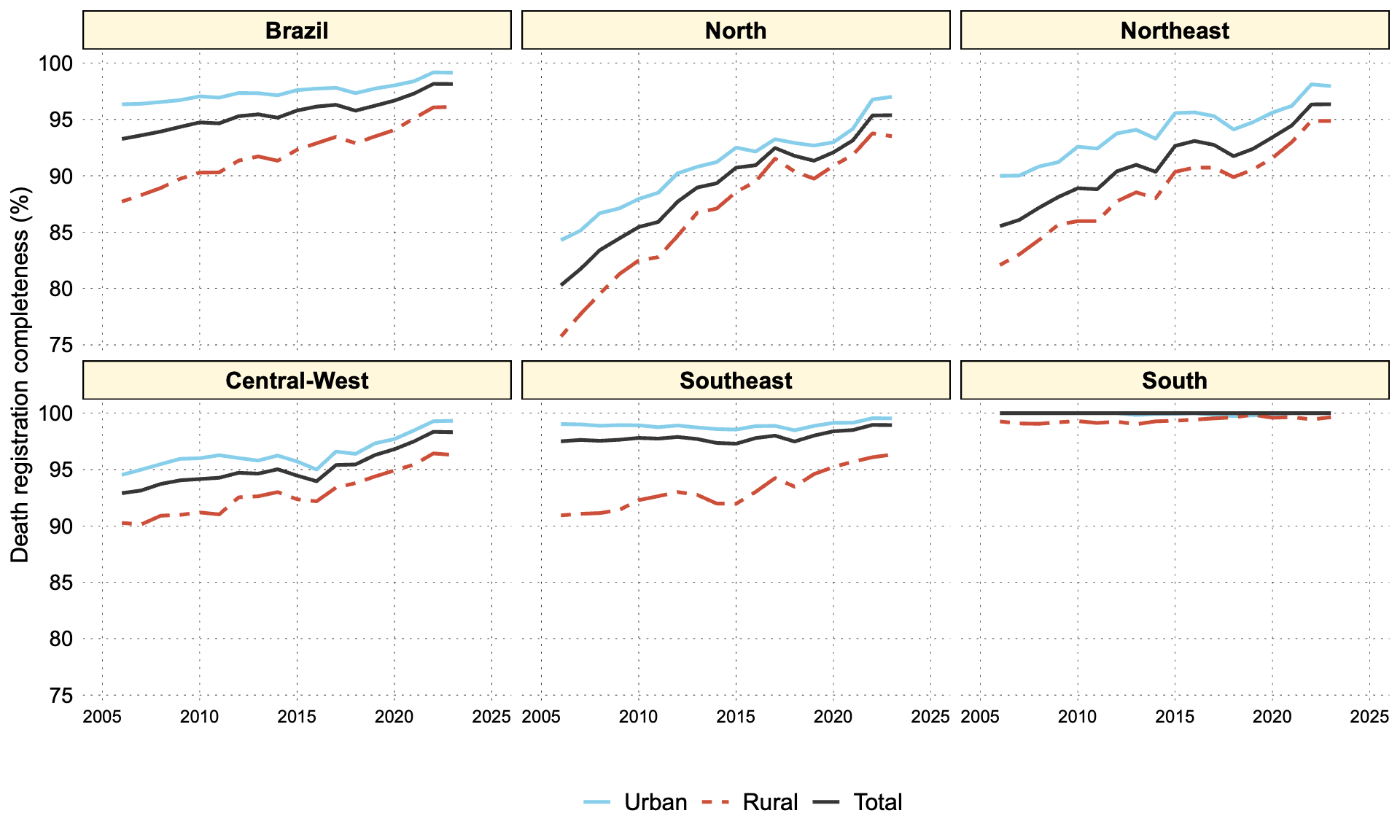
**

1. The datasets from the proactive search procedure can be found at https://svs.aids.gov.br/daent/acesso-a-informacao/acoes-e-programas/busca-ativa/indicadores-de-saude/cobertura/ [↑](#footnote-ref-1)
2. Information available at https://www.ibge.gov.br/estatisticas/sociais/populacao/26176-estimativa-do-sub-registro.html?=&t=resultados [↑](#footnote-ref-2)
