# Supplementary material for: The Emergence of an Urban Mortality Advantage in Brazil: An Assessment of Age and Causes-of-Death Contributions to the Urban–Rural Mortality Gap
Source: J Urban Health. 2026 Apr 23;103(3):587–99. doi: 10.1007/s11524-026-01076-0 (PMC13315069; doi:10.1007/s11524-026-01076-0)
Supplement: Supplementary file 4 — (DOCX 887 KB) [file 11524_2026_1076_MOESM4_ESM.docx]

# **Supplemental Material 4: Sensitivity analysis – alternative urban-rural classification**

In this supplementary material 4, we re-did our main analysis using a different definition to classify urban and rural areas; we define here urban areas as those municipalities with at least 50,000 inhabitants, and rural areas as those municipalities with less than 50,000 inhabitants in the 2022 census round. As we did in our analysis, here we also fix the definition as that from 2022 to previous years to make sure that we are comparing the same group of municipalities through time. As reported here in Figure S4.1 and Table S4.1, our results and conclusions are the same as those we had in the version we used in the main manuscript.

**Figure S4.1:** A) Life Expectancy between Ages 20 and 85 by Area, Region, and Sex. Brazil, 2006-2023. B) Urban-Rural Gap in Life Expectancy between Ages 20 and 85 by Area, Region, and Sex. Brazil, 2006-2023. Urban areas here are defined as municipalities with at least 50,000 inhabitants.


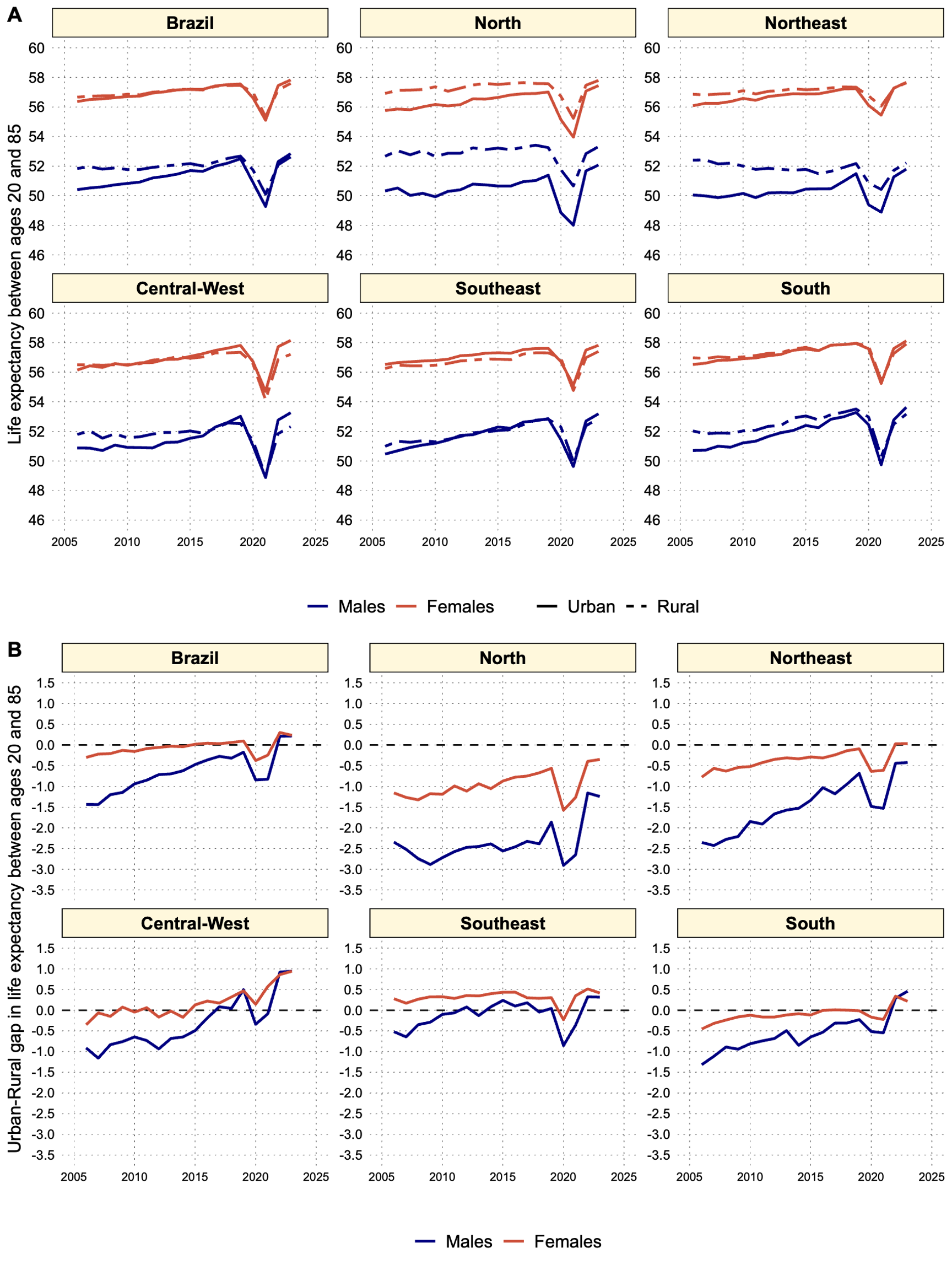


|  | Males | | | | Females | | | |
| --- | --- | --- | --- | --- | --- | --- | --- | --- |
|  | Urban | Rural | Total | Gap | Urban | Rural | Total | Gap |
| North | | | | | | | | |
| 2006 | 50.3 | 52.7 | 51.2 | -2.3 | 55.8 | 56.9 | 56.1 | -1.2 |
| 2012 | 50.4 | 52.9 | 51.3 | -2.5 | 56.2 | 57.3 | 56.5 | -1.1 |
| 2019 | 51.4 | 53.3 | 52.0 | -1.9 | 57.0 | 57.6 | 57.2 | -0.6 |
| 2020 | 48.9 | 51.8 | 49.8 | -2.9 | 55.2 | 56.7 | 55.6 | -1.6 |
| 2021 | 48.0 | 50.7 | 48.9 | -2.7 | 54.0 | 55.2 | 54.3 | -1.3 |
| 2022 | 51.7 | 52.8 | 52.1 | -1.2 | 57.1 | 57.5 | 57.2 | -0.4 |
| 2023 | 52.1 | 53.3 | 52.5 | -1.2 | 57.4 | 57.8 | 57.5 | -0.3 |
| Northeast | | | | | | | | |
| 2006 | 50.1 | 52.4 | 51.2 | -2.4 | 56.1 | 56.9 | 56.4 | -0.8 |
| 2012 | 50.2 | 51.9 | 51.0 | -1.7 | 56.7 | 57.1 | 56.9 | -0.3 |
| 2019 | 51.5 | 52.2 | 51.8 | -0.7 | 57.2 | 57.3 | 57.3 | -0.1 |
| 2020 | 49.4 | 50.9 | 50.1 | -1.5 | 56.1 | 56.7 | 56.4 | -0.6 |
| 2021 | 48.9 | 50.4 | 49.6 | -1.5 | 55.5 | 56.1 | 55.7 | -0.6 |
| 2022 | 51.3 | 51.7 | 51.5 | -0.4 | 57.3 | 57.2 | 57.3 | 0.0 |
| 2023 | 51.8 | 52.2 | 52.0 | -0.4 | 57.7 | 57.6 | 57.7 | 0.0 |
| Central-West | | | | | | | | |
| 2006 | 50.9 | 51.8 | 51.3 | -0.9 | 56.1 | 56.5 | 56.3 | -0.4 |
| 2012 | 50.9 | 51.8 | 51.2 | -0.9 | 56.7 | 56.8 | 56.7 | -0.2 |
| 2019 | 53.0 | 52.5 | 52.9 | 0.5 | 57.8 | 57.3 | 57.7 | 0.5 |
| 2020 | 51.0 | 51.4 | 51.2 | -0.3 | 56.7 | 56.6 | 56.7 | 0.1 |
| 2021 | 48.9 | 49.0 | 49.0 | -0.1 | 54.7 | 54.1 | 54.5 | 0.6 |
| 2022 | 52.8 | 51.8 | 52.5 | 0.9 | 57.7 | 56.9 | 57.5 | 0.9 |
| 2023 | 53.3 | 52.3 | 53.0 | 0.9 | 58.2 | 57.2 | 57.9 | 0.9 |
| Southeast | | | | | | | | |
| 2006 | 50.5 | 51.0 | 50.6 | -0.5 | 56.5 | 56.3 | 56.5 | 0.3 |
| 2012 | 51.7 | 51.6 | 51.8 | 0.1 | 57.1 | 56.8 | 57.1 | 0.4 |
| 2019 | 52.9 | 52.8 | 52.9 | 0.0 | 57.6 | 57.3 | 57.6 | 0.3 |
| 2020 | 51.5 | 52.3 | 51.7 | -0.9 | 56.7 | 57.0 | 56.8 | -0.2 |
| 2021 | 49.6 | 50.0 | 49.7 | -0.4 | 55.1 | 54.8 | 55.1 | 0.3 |
| 2022 | 52.7 | 52.4 | 52.7 | 0.3 | 57.5 | 57.0 | 57.4 | 0.5 |
| 2023 | 53.2 | 52.9 | 53.1 | 0.3 | 57.8 | 57.4 | 57.8 | 0.4 |
| South | | | | | | | | |
| 2006 | 50.7 | 52.0 | 51.3 | -1.3 | 56.5 | 57.0 | 56.7 | -0.5 |
| 2012 | 51.7 | 52.3 | 52.0 | -0.7 | 57.1 | 57.3 | 57.2 | -0.2 |
| 2019 | 53.3 | 53.5 | 53.4 | -0.2 | 57.9 | 58.0 | 58.0 | 0.0 |
| 2020 | 52.5 | 53.0 | 52.7 | -0.5 | 57.6 | 57.8 | 57.7 | -0.2 |
| 2021 | 49.7 | 50.3 | 50.0 | -0.5 | 55.2 | 55.5 | 55.4 | -0.2 |
| 2022 | 52.8 | 52.5 | 52.7 | 0.3 | 57.6 | 57.3 | 57.5 | 0.3 |
| 2023 | 53.6 | 53.2 | 53.5 | 0.5 | 58.1 | 57.9 | 58.1 | 0.2 |
| Brazil | | | | | | | | |
| 2006 | 50.4 | 51.8 | 50.9 | -1.4 | 56.4 | 56.7 | 56.5 | -0.3 |
| 2012 | 51.2 | 51.9 | 51.5 | -0.7 | 56.9 | 57.0 | 57.0 | -0.1 |
| 2019 | 52.5 | 52.7 | 52.6 | -0.2 | 57.6 | 57.5 | 57.5 | 0.1 |
| 2020 | 50.9 | 51.7 | 51.2 | -0.8 | 56.6 | 57.0 | 56.7 | -0.4 |
| 2021 | 49.3 | 50.1 | 49.6 | -0.8 | 55.1 | 55.3 | 55.2 | -0.2 |
| 2022 | 52.3 | 52.1 | 52.3 | 0.2 | 57.4 | 57.1 | 57.4 | 0.3 |
| 2023 | 52.8 | 52.6 | 52.8 | 0.2 | 57.8 | 57.6 | 57.8 | 0.2 |

**Table S4.1:** Life Expectancy between Ages 20 and 85 by Area, Region, and Sex. Brazil, 2006-2023. Urban areas here are defined as municipalities with at least 50,000 inhabitants.
